# Emotion dysregulation and eating disorder outcome: Prediction, change and contribution of self‐image

**DOI:** 10.1111/papt.12391

**Published:** 2022-03-25

**Authors:** Elin Monell, David Clinton, Andreas Birgegård

**Affiliations:** ^1^ Department of Clinical Neuroscience Centre for Psychiatry Research Karolinska Institute, and Stockholm Health Care Services Stockholm County Council Stockholm Sweden; ^2^ Department of Medical Epidemiology and Biostatistics Karolinska Institute Stockholm Sweden; ^3^ Institute for Eating Disorders Villa Sult Oslo Norway

**Keywords:** change scores, eating disorders, emotion dysregulation, mediation analysis, outcome, prediction, self‐image

## Abstract

**Objectives:**

Eating disorders (EDs) are severe disorders with unsatisfactory outcome. Emotion dysregulation and self‐image are suggested maintenance factors; this study examined emotion dysregulation as potential predictor and/or mechanism of change in relation to ED outcome, and associations between change in emotion dysregulation and self‐image in relation to outcome.

**Design:**

Registry data from initial and 1‐year follow‐up assessments for 307 patients with a wide range of EDs in specialized ED treatment were used.

**Methods:**

Initial and change (∆) in emotion dysregulation were examined as predictors of 1‐year outcome. Direct and indirect associations between ∆emotion dysregulation and ∆self‐image as either independent variable or mediator in relation to ∆ED psychopathology as dependent were also examined.

**Results:**

Higher initial emotion dysregulation was weakly associated with higher follow‐up ED psychopathology, but not remission, while relative increase in emotion dysregulation was associated with both higher follow‐up psychopathology and increased risk of still having a diagnosis. Change in emotion dysregulation primarily had an indirect effect (through change in self‐image), while change in self‐image had a direct effect, on change in ED psychopathology improvement (such that improvement in one was associated with improvement in the other).

**Conclusions:**

Results identify emotion dysregulation as a potential mechanism of change in relation to ED outcome. However, this association was mainly mediated by change in self‐image. Results indicate that, in order to improve emotion regulation as a means to reduce ED psychopathology, improving self‐image is essential.

**Practitioner points**

- Emotion dysregulation and negative self‐image are central themes in eating disorders (EDs) with implications for ED maintenance and outcome.
- Improvements in emotion regulation during treatment shows associations with improvements in ED symptoms, but mainly when improvements in self‐image are taken into account.
- Helping patients to increase the ability to treat oneself and one's emotions with acceptance rather than blame, even in the presence of unwanted or undifferentiated emotions, may increase their chances of remission from the ED.

## INTRODUCTION

Eating disorders (EDs) are complex psychiatric disorders characterized by preoccupation with eating, shape and weight, dietary restraint, binge eating and/or compensatory behaviours (American Psychiatric Association, 2013). They are associated with decreased interpersonal functioning, life disruptions, high somatic and psychiatric comorbidity and increased mortality (Schaumberg et al., 2017). Relapses are common and about one fifth develop a chronic illness (Keel & Brown, 2010; Keel et al., 2005). Also, a substantial number of recovered individuals still have residual ED symptoms, lower social functioning and lower psychological well‐being (Tomba et al., 2019). Therefore, the identification of reliable and clinically targetable predictors and mechanisms of change is crucial to improve outcome. In this context, *predictors* define initial factors predicting outcome, while *mechanisms of change* (often examined as change scores and/or within mediation models) define psychological processes, affected by specific techniques and the therapeutic relationship, impacting outcome (Petrik & Cronin, 2014; Vall & Wade, 2015). Previous research suggests emotion dysregulation and self‐image as such potential mechanisms (Monell et al., 2020), but their interaction in relation to ED outcome has not been examined.

Emotion regulation and self‐image are developed in ongoing interactions with primary attachment figures, starting in early childhood but continually developing throughout life (Mikulincer & Shaver, 2019; Thompson, 2019). Initially, all affective states are recognized, labelled and regulated by caregivers, laying the foundation for socio‐emotional development and functioning. In parallel, the sense of self is developed. Emotion‐related interactions are *introjected*, forming self‐image as a template for future self‐directed evaluations and behaviours (Benjamin, 2018). Self‐image is defined in terms of both affective valence and aspects of control, concretized as positive self‐image aspects such as self‐affirmation, ‐love and/or ‐protection on the one hand, and negative self‐image aspects such as self‐blame, ‐attack and/or ‐neglect on the other (Benjamin, 2018). Rather than being a descriptive construct regarding self‐worth (as in self‐esteem) or own traits/competencies (as in self‐confidence), interpersonal self‐image explicitly operationalizes self‐directed behaviours, that is, what one does to, or for, oneself. Self‐image tends to stabilize over time, in part as it directs how interactions with others are perceived and interpreted (Sullivan, 1953), while one also tends to behave in ways that evoke responses from others in line with one's present self‐image (Benjamin, 2018). Emotion dysregulation, conceptualized as difficulties in emotional awareness, clarity and acceptance, and difficulties managing emotional distress (Gratz & Roemer, 2004), presumably develop through the interaction between individual vulnerabilities and invalidating responses from others (Calkins et al., 2019; Linehan, 1993). As described by Linehan (1993), undifferentiated and unmanageable emotional arousal may increase usage of dysfunctional emotion regulation strategies and reduce the ability to adaptively communicate emotions to others, increasing the risk of invalidating responses. Such experiences are also assumed to affect self‐image, increasing negative and decreasing positive aspects (Benjamin, 2018).

The impact of emotional difficulties has long been recognized in EDs. A main contributor was Hilde Bruch, who emphasized difficulties in emotional processing and understanding in her work on restrictive EDs (i.e. mainly anorexia nervosa [AN]; Bruch, 1978, 1982). More recent theories posit emotional difficulties and associated traits as central factors in ED aetiology and maintenance (Haynos & Fruzzetti, 2011; Pearson et al., 2015; Treasure & Schmidt, 2013). The role of emotion dysregulation in ED maintenance has received increased attention in both research and treatment (Linardon et al., 2017; Prefit et al., 2019; Schaefer et al., 2020), including the notion of behavioural ED symptoms as maladaptive strategies for emotion regulation. Negative and critical ways of evaluating oneself is also a central feature in EDs. Diagnostic criteria for both AN and bulimia nervosa (BN) highlight the role of weight and shape for self‐worth (APA, 2013), and (struggle for) control over eating is also central for self‐evaluation. Accordingly, traits such as self‐esteem, self‐control and self‐criticism are highlighted in both theoretical and empirical work in EDs (e.g. Fairburn et al., 2009). Additionally, EDs imply doing potentially dangerous things to oneself (starvation, eating until being sick, painful purging, etc.). That is, negative attitudes towards the self in EDs are not only represented by passive evaluations of the self or one's body but also imply a wide range of negative, self‐directed behaviours tightly connected with symptoms. As such, by capturing central aspects of how one generally thinks of and behaves towards oneself (i.e. incorporating both self‐directed evaluations and behaviours), the self‐image construct is particularly relevant in EDs with implications for both development and maintenance of symptoms as well as general functioning.

Both emotion dysregulation and negative self‐image, particularly in terms of lower self‐affirmation, lower self‐love and higher self‐blame, have been associated with higher concurrent ED pathology (Forsén Mantilla & Birgegård, 2015; Monell et al., 2018; Prefit et al., 2019), but longitudinal studies examining their associations with ED outcome are rarer. Initial emotion dysregulation has shown some associations with reduced behavioural symptoms after treatment in patients with EDs characterized by binge eating (i.e. mainly BN and binge eating disorder [BED]; Accurso et al., 2016; Anderson et al., 2020), as well as some improvements in ED psychopathology in BN (Accurso et al., 2016), although not consistently (MacDonald & Trottier, 2019; MacDonald et al., 2017). As such, the predictive value of emotion dysregulation is unclear. Reductions in emotion dysregulation have more consistently been associated with both remission and reduced ED psychopathology after treatment in a range of EDs, as well as reduced behavioural symptoms in BN and BED (e.g. Hazzard et al., 2020; MacDonald & Trottier, 2019; MacDonald et al., 2017; Mallorquí‐Bagué et al., 2018; Peterson et al., 2017; Rowsell et al., 2016), suggesting emotion dysregulation as a potential *mechanism of change* influencing outcome. However, treatment in existing studies (cognitive behavioural therapy [CBT] with or without some emotion regulation add‐on) does not represent overall ED treatment settings. Instead, ED treatment as usual (TAU) includes a multitude of interventions and various psychotherapeutic approaches. Also, no study followed patients up to 12 months. As to self‐image, higher negative and lower positive aspects have shown strong associations with poorer 1‐ and 3‐year ED outcomes across ED diagnoses (Birgegård et al., 2009; Björck et al., 2007; Forsén Mantilla et al., 2019). Further, improvements in self‐image (i.e. reductions in negative and/or increases in positive aspects) have been associated with improvements in ED psychopathology in BN, and with reductions in binge eating frequency in BED (Hazzard et al., 2020; Peterson et al., 2017). Thus, self‐image may represent both a *predictor* and *mechanism of change* of ED outcomes, although the evidence for the latter is sparser.

Several theoretical models of ED aetiology and maintenance include both self‐ and emotion‐related factors (Pennesi & Wade, 2016), although few studies have examined their interactions. When examined concurrently within mediation models in a registry‐based study across EDs, and in university women, more negative self‐image was strongly associated with higher ED psychopathology regardless of emotion dysregulation levels (Monell et al., 2015, 2018). Emotion dysregulation on the other hand mainly had an indirect effect on ED psychopathology in these studies; higher emotion dysregulation mediated by more negative self‐image was associated with higher ED psychopathology. This suggests self‐image as a mechanism whereby emotion dysregulation affects ED psychopathology. That is, greater emotion dysregulation seems to go hand in hand with more pathological self‐directed evaluations and behaviours, which in turn may increase negative appearance‐related evaluations and efforts to control the body. For example, experiencing one's emotions as unwanted, unclear and unmanageable may elicit negative self‐directed thoughts and behaviours, which leads to negatively targeting the body as something more concrete and ‘controllable’. Thus, to reduce the effect of emotion dysregulation on ED psychopathology, the findings in Monell et al. (2015, 2020) suggest the importance of helping patients respond to themselves with acceptance rather than harsh blame, even in the presence of unwanted or undifferentiated emotions. Associations between these self‐directed evaluations and behaviours and ED pathology may also be more clearly experienced by patients, while connections to their (often poorly understood) emotions might seem both distal and diffuse. Subjectively, negative self‐image may also more clearly both increase and overlap ED symptoms. Self‐attack, ‐blame and ‐neglect may be experienced as negative self‐evaluation harshly demanding increased restraint, purging or exercise, regardless of one's actual needs; lack of self‐acceptance, ‐love and ‐protection as denying oneself rest, food enjoyment or being uncomfortable in one's own body (e.g. Espeset et al., 2012). Negative self‐image may also approximate the experience of the controlling and critical ‘ED voice’ (Aya et al., 2019). However, as these findings were based on cross‐sectional data, temporal associations are speculative.

In summary, knowledge of the predictive value of emotion dysregulation for ED outcome across EDs is limited, and even less is known about the effect of emotion dysregulation and outcome after TAU, and over longer follow‐ups. Additionally, there is to our knowledge no work examining the interplay between self‐image and emotion dysregulation in relation to ED outcome, which could inform theories of ED maintenance and suggest a framework for effectively targeting self‐image and emotion dysregulation. This study therefore sought to clarify the role of emotion dysregulation as a predictor, mechanism of change, or both in relation to 1‐year outcome in patients with a wide range of EDs receiving TAU. It also sought to examine pathways whereby change in emotion dysregulation and self‐image might influence change in ED psychopathology over 1 year. Specifically, the study aimed to (1) predict outcome (both remission and ED psychopathology) by initial emotion dysregulation, and by change in emotion dysregulation, from initial registration to follow‐up; and (2) examine two exploratory models of direct and indirect effects (i.e. mediation models), with change from initial registration to follow‐up in emotion dysregulation and self‐image as either independent or mediating variables in relation to change in ED psychopathology.

## METHODS

### Participants

Study participants were patients with initial AN restrictive or binge/purge subtype (AN‐R; AN‐BP), BN, BED and other specified feeding and EDs (OSFED) in Swedish specialized ED treatment drawn from the nationwide Stepwise clinical database (Birgegård et al., 2010). The sample consisted of 307 patients aged 16–58 years with relevant study data (see Procedure) at both initial and follow‐up assessment (detailed sample description in Table 1). At follow‐up, treatment had been terminated for almost half of participants. Between assessment points, participants had usually received psychotherapy and/or supportive therapy in combination with other interventions (e.g. psychoeducation, physical/dietary counselling, physiotherapeutic treatment and assisted eating). More than half had received psychotherapy, mostly CBT with or without additional psychotherapy (detailed treatment characteristics in Table S1).

**TABLE 1:** Descriptive statistics at initial registration and 1‐year follow‐up for the whole sample and per initial ED group

	All	AN‐R	AN‐BP	BN	BED	OSFED
	*M* (*SD*)	*M* (*SD*)	*M* (*SD*)	*M* (*SD*)	*M* (*SD*)	*M* (*SD*)
Initial registration						
*N*	307	50	15	104	19	119
Females (*n*; %)	292 (95.1%)	48 (96%)	15 (100%)	97 (93.3%)	19 (100%)	113 (95.0%)
Age	24.47 (8.25)	21.68 (7.89)	22.80 (6.75)	26.63 (8.29)	31.53 (10.02)	22.83 (7.17)
ED duration	8.23 (8.09)	6.04 (7.30)	6.33 (7.72)	10.55 (8.25)	13.89 (10.14)	6.45 (7.06)
BMI	21.91 (6.03)	16.30 (1.44)	16.23 (1.21)	24.86 (5.80)	33.77 (6.54)	20.52 (2.79)
CPRS anxiety	9.78 (4.26)	8.27 (4.37)	11.60 (4.03)	10.23 (4.41)	9.75 (3.22)	9.65 (4.17)
EDE‐Q global	3.90 (1.12)	3.31 (1.27)	3.87 (1.21)	4.17 (.98)	3.53 (1.00)	3.97 (1.10)
SASB affiliation	−14.36 (33.04)	−5.05 (37.51)	−13.45 (38.31)	−18.50 (34.33)	−18.27 (25.05)	−14.14 (29.90)
DERS total	101.80 (25.45)	98.68 (28.42)	100.00 (29.99)	103.88 (23.32)	96.58 (18.83)	102.37 (26.37)
Follow‐up registration						
No ED (*n*; %)	172 (52.3%)	22 (44.0%)	5 (33.3%)	62 (59.6%)	16 (84.2%)	59 (49.6%)
AN‐R (*n*; %)	25 (7.6%)	18 (31.6%)	2 (11.1%)	0 (0.0%)	0 (0.0%)	5 (4.0%)
AN‐BP (*n*; %)	5 (1.5%)	1 (1.8%)	3 (16.7%)	1 (0.9%)	0 (0.0%)	0 (0.0%)
BN (*n*; %)	27 (8.2%)	1 (1.8%)	0 (0.0%)	22 (19.8%)	0 (0.0%)	4 (3.2%)
BED (*n*; %)	11 (3.3%)	0 (0.0%)	0 (0.0%)	7 (6.3%)	2 (10.5%)	2 (1.6%)
OSFED (*n*; %)	76 (23.1%)	12 (21.1%)	7 (38.9%)	15 (13.5%)	0 (0.0%)	42 (33.9%)
UFED (*n*; %)	13 (4.0%)	1 (1.8%)	1 (5.6%)	2 (1.8%)	1 (5.3%)	8 (6.5%)
BMI	23.13 (5.62)	18.89 (2.52)	18.59 (1.94)	25.12 (5.86)	34.20 (5.78)	21.98 (2.96)
CPRS anxiety	7.61 (4.47)	6.66 (4.13)	10.00 (4.60)	8.05 (4.80)	7.78 (4.06)	7.19 (4.22)
EDE‐Q global	2.25 (1.58)	1.91 (1.59)	3.13 (1.62)	2.26 (1.59)	2.15 (1.25)	2.28 (1.58)
SASB affiliation	18.84 (44.93)	23.10 (48.90)	−0.31 (45.43)	18.20 (46.11)	19.78 (38.96)	19.91 (43.07)
DERS total	87.60 (29.58)	81.58 (26.88)	113.50 (22.71)	90.18 (29.10)	71.70 (19.33)	87.61 (31.50)
DERS valid *N*	158	24	6	57	10	61

Abbreviations: AN‐BP, anorexia nervosa binge/purge subtype; AN‐R, anorexia nervosa restrictive subtype; BED, binge eating disorder; BMI, body mass index; BN, bulimia nervosa; CPRS, Comprehensive Psychopathological Rating Scale; DERS, Difficulties in Emotion Regulation Scale; ED, eating disorder; EDE‐Q, Eating Disorder Examination Questionnaire; OSFED, other feeding and eating disorders; SASB, Structural Analysis of Social Behaviors.

### Procedure

Stepwise includes patients of all ages entering treatment since 2005. Inclusion criteria are referral to a treatment unit, a DSM‐IV ED (APA, 2000), and treatment unit intent‐to‐treat. Stepwise initial and 1‐year follow‐up assessment includes both mandatory and optional semi‐structured interviews, clinician‐ratings and self‐ratings. Clinicians chose the optional assessments and their reasons for doing so are not recorded. For more information on Stepwise procedures, see Birgegård et al., 2010. Follow‐up attrition is high (~40–70%), with a trend of higher attrition the longer Stepwise has been running (Rania et al., 2020).

Study data were extracted dated 19 February 2020 including cases with initial registrations between 07 April 2014 (date of emotion dysregulation measure inclusion into Stepwise) and 20 January 2020. Extraction also included available follow‐up data. Initial exclusions (empty registrations, patients <16 year of age, no ED after assessment) left 5335 potential cases. Secondary exclusions were no general research consent (*n* = 214), no self‐ratings (*n* = 239), ED not otherwise specified (EDNOS) not relevant for this study (‘other’ *n* = 110; ‘chewing and spitting’ *n* = 83), and duplicate/multiple registrations (first registration kept; *n* = 114); this left 4577 patients. The DSM‐IV diagnoses were then recategorized post‐hoc using DSM‐5 criteria. Lastly, patients without DERS ratings at initial registration (*n* = 2588) and patients without complete follow‐ups (both diagnostic assessment and ED pathology self‐ratings at follow‐up; *n* = 1682) were excluded, leaving 307 participants in the final sample. When excluding participants that had not yet passed the follow‐up assessment window (*n* = 336), attrition was 79.9%. For the remainder, 506 patients had no follow‐up due to their declining this assessment (37%), not being eligible at units at a later timepoint (34%), or could not be reached (29%), while no reason for attrition was recorded for 840 patients (attrition for these was likely due to administrative reasons and/or lack of time at units).

### Measures

*The Difficulties in Emotion Regulation Scale* (DERS; Gratz & Roemer, 2004) measures emotion dysregulation through 36 items scored 1–5. Items form one Total score and six subscales. The Total score (sum of subscale sums), where higher scores indicate greater difficulties, was used in the analyses. The DERS is optional in Stepwise assessments; 158 participants had both initial and follow‐up DERS. It has good overall psychometric properties (Gratz & Roemer, 2004), as has the Swedish version (Monell et al., 2021; Nordgren et al., 2020). Internal consistency (Cronbach's alpha; α) was good‐excellent for initial (αs = .803–.905; mean α = .867) and good‐excellent for follow‐up subscales forming the Total score (αs = .887–.924; mean α = .906).

*The Eating Disorder Examination Questionnaire*, version 4.0 (EDE‐Q; Fairburn & Beglin, 1994) measures ED pathology in the last 28 days through 36 items scored 0–6. Items form one Global score and four subscales; additional items also measure behavioural symptoms. The Global score (mean of subscale means), where higher scores indicate greater ED psychopathology, was used in the analyses. The EDE‐Q is mandatory in Stepwise assessments. It has good psychometric properties (Luce & Crowther, 1999; Mond et al., 2008), as has the Swedish version (Welch et al., 2011). Internal consistency was questionable‐good for initial (αs = .637–.878; mean α = .770) and good‐excellent for follow‐up subscales forming the Global score (αs = .874–.929; mean α = .880).

*The Structural Analysis of Social Behaviors* (SASB), Intrex version, Introject (Benjamin, 2000) measures self‐image through 36 items scored 0–100 with 10‐point increments. Items form eight clusters located in a circumplex organized by the horizontal Affiliation (ranging from self‐love to self‐attack) and vertical Autonomy axes (ranging from self‐emancipation to self‐control). The Affiliation score was used in the analyses. It is a weighted summary of the Affiliation cluster scores (i.e. positive clusters self‐affiliation, ‐love and ‐protection, and negative clusters self‐blame, ‐attack and ‐neglect) ranging between −100 and 100; scores below zero reflect negative self‐image, scores above reflect positive. The SASB is mandatory in Stepwise assessments and has good psychometric properties (Benjamin, 2000). Internal consistency was questionable‐good for initial (αs = .667–.818; mean α = .748) and good‐excellent for follow‐up Affiliation cluster scores (αs = .798–.920; mean α = .869).

*Comprehensive Psychopathological Rating Scale*, *Self*‐*rated form*, *Affective scales* (CPRS‐S‐A; Svanborg & Åsberg, 1994) measures emotional symptoms the past 3 days through 19 items scored 0–3 with 0.5‐point increments. The Anxiety subscale was used as a covariate (see Statistical analysis). The CPRS is mandatory in Stepwise assessments for patients ≥18 years. It has good psychometric properties (Svanborg & Åsberg, 1994). Internal consistency was fair for initial and good for follow‐up anxiety (αs = .795 and .846).

*The Structured Eating Disorder Interview* (SEDI; de Man Lapidoth & Birgegård, 2010) is a semi‐structured interview used to diagnose EDs according to DSM‐IV criteria, developed for and used within the Stepwise system. Diagnostic information was additionally used for follow‐up remission status; patients no longer fulfilling diagnostic criteria for any ED were categorized as having no ED. The SEDI is mandatory in Stepwise assessments.

### Statistical analysis

Analyses were performed using SPSS‐25 for Mac. To reduce the risk of Type‐I errors, significance level was set to *p* < .01. Paired samples *t*‐tests and effect size (Cohen's *d*; small >.02; medium >.50; large >.80) examined overall change from initial to follow‐up registration in DERS Total, EDE‐Q Global, and SASB Affiliation. Standardized regression residuals of DERS Total, EDE‐Q Global and SASB Affiliation, used as change scores (∆), were computed by regressing follow‐up values on initial values. ∆SASB was then reversed so that it could be interpreted like ∆EDE‐Q and ∆DERS Total (i.e. higher scores indicate higher severity). For simplicity, these ∆variables will be described in relation to the regression line, that is, relative to the sample norm change rather than absolute scale scores. Thus, an ‘increase’ suggests a higher score than expected by the regression model, and ‘decrease’ a lower one, whether or not the absolute follow‐up score is higher or lower than at initial assessment. This is because the present aim revolves around associations rather than absolute outcome. Prior to analyses, multivariate outliers in each set of independent variables were detected through computation of Mahalanobis’ distances. Models were run with and without outliers; results excluding outliers were highly concordant and therefore not reported.

Regression models in the entire sample examined 1‐year outcome, defined as follow‐up ED psychopathology and ED remission status (logistic regression; 0 = no ED, 1 = ED left) respectively. Predictors were (a) initial DERS Total and (b) ∆DERS Total. All models were rerun with covariates (1) including initial EDE‐Q Global, ED duration and BMI (defining initial severity), and (2) additionally including CPRS Anxiety (run separately as participants <18 years lack CPRS). Anxiety was chosen over depression as a covariate as depression and anxiety were strongly correlated (*r* = .78^***^) and anxiety showed the strongest associations with both outcomes (examined by stepwise regression with both subscales predicting each outcome, results not shown). Exploratory regression‐based analyses of direct and indirect effects (mediation) examined how changes in emotion dysregulation and self‐image influenced change in ED psychopathology over the year, using change scores. Two models were examined with either ∆DERS Total or ∆SASB Affiliation score as independent (*X*) or mediating variable (*M*) and ∆EDE‐Q Global as dependent variable (*Y*). Analyses were performed using PROCESS macro 3.5, Model 4 (Hayes, 2018) with 99% confidence intervals (CI) based on 10,000 bootstrap samples for statistical inference of the indirect effect.

#### Sample representativeness

Differences between study participants and those excluded at various levels in initial and follow‐up study variables (when available), age, ED duration, BMI, gender, initial diagnoses and treatment characteristics were examined by univariate analysis of variance and chi‐square per diagnostic subgroup. Effect sizes ($\eta_{\text{partial}}^{2}$, Phi coefficients [Φ]) were computed for significant differences (small $\eta_{\text{partial}}^{2}$ > .01/Φ > .10, medium $\eta_{\text{partial}}^{2}$ > .06/Φ > .30, large $\eta_{\text{partial}}^{2}$ > .14/Φ > .50). Differences at *p* < .05 with ≥ small effect sizes were considered meaningful.

In all potential participants (*n* = 4577), neither those with (*n* = 1989) or without (*n* = 2588) initial DERS nor those with follow‐up assessments with (*n* = 307) or without (*n* = 467*) initial DERS differed meaningfully, except in two groups with follow‐up assessments. In OSFED, patients with initial DERS were slightly younger with shorter ED duration and lower BMI, while in BN, those with DERS had lower initial self‐image (all small effects). Lastly, those with initial DERS more often received psychotherapy (as opposed to primarily supportive therapy), these were also more often CBT‐based (small effects; only examined in the whole sample). Participants with initial DERS did not differ meaningfully depending on if they had follow‐ups (*n* = 307) or not (*n* = 1346^1^), except that AN‐R and OSFED with follow‐ups had lower anxiety and BMI, respectively (small effects), and BED with follow‐ups had lower DERS Impulse (medium effect). In the final sample (*N* = 307), participants did not differ meaningfully depending on whether they had follow‐up DERS (*n* = 158) or not (*n* = 149) except in AN‐R. Here, those with follow‐up DERS had lower initial DERS Non‐Acceptance (large effect) and lower DERS Strategies and anxiety (medium effects).

## RESULTS

At 1‐year follow‐up, more than half of patients were in remission, with highest remission rate in BED and lowest in AN‐BP (Table 1). Participants significantly improved in mean ratings of ED psychopathology (large effect), self‐image and emotion dysregulation (medium effects; Table 2). Diagnostic subgroup changes were similar to the overall changes, except in AN‐BP with higher mean emotion dysregulation at follow‐up.

**TABLE 2:** Change in main variables from initial registration to 1‐year follow‐up (raw change; post–pre)

	All				AN‐R		AN‐BP		BN		BED		OSFED	
	*M* (*SD*)	*t*	*p*	*d*	*M* (*SD*)	*d*	*M* (*SD*)	*d*	*M* (*SD*)	*d*	*M* (*SD*)	*d*	*M* (*SD*)	*d*
EDE‐Q global	−1.65 (1.49)	−19.46	<.001	−1.25	−1.40 (1.43)	−0.98	−0.74 (1.07)	−0.69	−1.91 (1.43)	−1.33	−1.37 (1.65)	−.083	−1.69 (1.53)	−1.11
SASB affiliation	33.27 (39.35)	14.76	<.001	0.71	28.69 (41.03)	0.70	13.13 (28.78)	0.46	36.70 (38.02)	0.97	38.05 (42.82)	0.89	33.93 (40.05)	0.85
DERS total	−15.16 (27.77)	−7.40	<.001	−0.59	−8.25 (21.43)	−0.38	8.33 (20.00)	0.42	−17.16 (23.72)	−0.72	−28.50 (28.27)	−1.01	−16.13 (27.82)	−0.58

Note

Change in the whole sample examined by paired samples *t*‐test and effect size (Cohen's *d* for paired *t*‐tests). Effect sizes also computed for diagnostic subgroup change.

Abbreviations: AN‐BP, anorexia nervosa binge/purge subtype; AN‐R, anorexia nervosa restrictive subtype; BED, binge eating disorder; BN, bulimia nervosa; DERS, Difficulties in Emotion Regulation Scale; EDE‐Q, Eating Disorder Examination Questionnaire; OSFED, other feeding and eating disorders; SASB, Structural Analysis of Social Behaviors.

### Prediction of follow‐up ED psychopathology and remission

Higher initial emotion dysregulation was weakly associated with higher follow‐up ED psychopathology. However, this association did not remain in adjusted models. Initial emotion dysregulation was not associated with remission status (Table 3). Change in emotion dysregulation (i.e. ∆DERS Total) was strongly associated with follow‐up ED psychopathology, such that deterioration (i.e. positive scores/relative increase) indicated higher follow‐up psychopathology (Table 3). This association remained significant when adjusted for initial clinical severity and when additionally adjusted for anxiety. ∆DERS Total was also associated with remission status, such that deterioration indicated an increased risk of still having a follow‐up ED (Table 3). This association remained in adjusted models.

**TABLE 3:** Prediction of ED psychopathology at follow‐up and of remission (0 = no ED; 1 = still ED) at follow‐up, respectively, by initial DERS Total score (*N* = 307) and change in DERS Total score (∆Total score; standardized regression residuals; *n* = 158)

Variables	Unadjusted					Adjusted (1)					Adjusted (2) *n* = 243				
	*B* (se)	*beta*	*t*	*p*	*R* ^2^	*B* (se)	*beta*	*t*	*p*	*R* ^2^	*B* (se)	*beta*	*t*	*p*	*R* ^2^
Outcome = follow‐up EDE‐Q Global															
Initial DERS Total	.011 (<.01)	.171	3.01	.003	.029	−.002 (<.01)	−.028	−.480	.632	.193	−.005 (<.01)	−.089	−1.24	.217	.213
ED duration						.016 (.01)	.084	1.545	.123		.011 (.01)	.059	0.99	.322	
BMI						−.004 (.01)	−.017	−.303	.762		−.006 (.02)	−.025	−0.42	.678	
Initial EDE‐Q						.624 (.08)	.444	7.655	<.001		.588 (.10)	.402	5.75	<.001	
CPRS anxiety											.057 (.03)	.155	2.06	.040	
∆DERS Total	.866 (.11)	.531	7.82	<.001	.282	.871 (.09)	.534	9.427	<.001	.513	.847 (.11)	.522	7.99	<.001	.500
ED duration						.009 (.01)	.046	.798	.426		.006 (.01)	.032	0.49	.628	
BMI						.013 (.02)	.048	.834	.405		.012 (.02)	.047	0.71	.479	
Initial EDE‐Q						.680 (.08)	.475	8.371	<.001		.703 (.11)	.489	6.19	<.001	
CPRS anxiety											−.007 (.03)	−.018	−0.22	.826	

Variables	Unadjusted							Adjusted (1)							Adjusted (2) *n* = 243						
	*B* (*se*)	*Wald*	*p*	*OR*	*CI* LL	*CI* UL	*R* ^2^	*B* (*se*)	*Wald*	*p*	*OR*	*CI* LL	*CI* UL	*R* ^2^	*B* (*se*)	*Wald*	*p*	*OR*	*CI* LL	*CI* UL	*R* ^2^
Outcome = remission status																					
Initial DERS Total	−.004 (<.01)	0.82	.366	.996	.99	1.00	.003	−.013 (.01)	6.13	.013	.987	.98	1.00	.101	−.021 (.01)	8.90	.003	.980	.97	.99	.129
ED duration								.032 (.02)	3.96	.047	1.033	1.00	1.07		.034 (.02)	3.61	.057	1.034	1.00	1.07	
BMI								−.121 (.03)	19.19	<.001	.886	.84	.94		−.115 (.03)	16.01	<.001	.892	.84	.94	
Initial EDE‐Q								.428 (.13)	11.37	.001	1.534	1.20	1.97		.314 (.16)	3.89	.049	1.370	1.00	1.87	
CPRS anxiety															.107 (.04)	6.15	.013	1.113	1.02	1.21	
∆DERS Total	.882 (.20)	18.90	<.001	2.416	1.62	3.60	.139	.896 (.21)	17.46	<.001	2.451	1.61	3.73	.228	1.136 (.01)	18.07	<.001	3.113	1.84	5.25	.285
ED duration								.040 (.02)	3.17	.075	1.041	1.00	1.09		.047 (.02)	3.57	.059	1.048	1.00	1.10	
BMI								−.130 (.04)	8.80	.003	.878	.81	.96		−.130 (.05)	7.24	.007	.878	.80	.97	
Initial EDE‐Q								.423 (.17)	6.23	.013	1.527	1.10	2.13		.567 (.25)	5.34	.021	1.763	1.09	2.85	
CPRS anxiety															−.026 (.06)	.17	.683	.974	.86	1.11	

Note

Models run without covariates (unadjusted), with initial ED severity (ED duration, BMI, initial EDE‐Q Global; adjusted 1), and with initial ED severity and initial anxiety (adjusted 2). For dichotomous models: positive OR = increased risk of still being ill; negative OR = decreased risk of still being ill.

Abbreviations: BMI, body mass index; CPRS, Comprehensive Psychopathological Rating Scale; DERS, Difficulties in Emotion Regulation Scale; ED, eating disorder; EDE‐Q, Eating Disorder Examination Questionnaire (Global Score).

### Direct and indirect associations between 1‐year change in emotion dysregulation, self‐image and ED psychopathology

**FIGURE 1:**
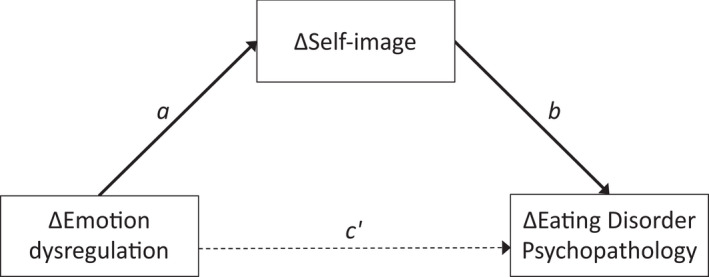
Visualization of version 1 mediation result depicting the indirect effect of less favourable change in emotion dysregulation on less favourable change in eating disorder psychopathology, through less favourable change in self‐image

All change variables were significantly associated with each other when examined separately (paths *a* and *c*, Table 4); change in either emotion dysregulation or self‐image was significantly associated with change in ED psychopathology, such that deterioration in one was associated with deterioration in the other or improvement associated with improvement (Table 4, total effects; indicated by a positive association between ∆DERS and ∆EDE‐Q in version 1 and a positive association between ∆SASB and ∆EDE‐Q in version 2). Change in emotion dysregulation was also significantly associated with change in self‐image (path *a*; indicated by a positive association between ∆SASB and ∆DERS). Mediator inclusion in each model showed differentiating direct and indirect effects. In version 1, inclusion of ∆SASB as a mediator diminished the association between ∆DERS and ∆EDE‐Q considerably; there was no longer a significant direct effect. Instead, there was an indirect effect of ∆DERS on ∆EDE‐Q through ∆SASB. *That is, change in emotion dysregulation was indirectly associated with change in ED psychopathology through change in self‐image* (Figure 1). In version 2, inclusion of ∆DERS as a mediator only slightly decreased the association between ∆SASB and ∆EDE‐Q; there was still a significant direct effect (path *c*´) independent of ∆DERS, and no evidence for an indirect effect through emotion dysregulation (path *ab*) as CI included zero. *That is, change in self‐image was associated with change in ED psychopathology regardless of change in emotion dysregulation*.

**TABLE 4:** Exploratory mediation models using either change in DERS Total or SASB Affiliation as independent (*X*) or mediator variable (*M*) in relation to change in EDE‐Q Global Score as dependent variable (*Y*)

	Version 1					Version 2				
	*X* = ∆DERS; *M* = ∆SASB; *Y* = ∆EDEQ					*X* = ∆SASB; *M* = ∆DERS; *Y* = ∆EDEQ				
	β	*SE*	*p*	99% CI	*R* ^2^	β	*SE*	*p*	99% CI	*R* ^2^
Total effect										
Path *c*	.606	.06	<.001		.361	.797	.06	<.001		.560
Path *a*	.645	.06	<.001		.464	.719	.06	<.001		.464
Path *b*	.674	.08	<.001		.575	.171	.07	.019		.575
Direct effect										
Path *c*´	.171	.07	.019			.**674**	.**08**	**<.001**		
Indirect effect										
Path *ab*	.**435**	.**06**		.**274 –.610**		.123	.06		−.287 –.029	

Note

Standardized regression residuals used as change scores. *N* = 158. Bold indicate significant direct effects (*c*´) and indirect effects (*ab*) with a 99% CI completely above or below zero.

Abbreviations: DERS, Difficulties in Emotion Regulation Scale; ED, eating disorder; EDE‐Q, Eating Disorder Examination Questionnaire; SASB, Structural Analysis of Social Behaviors.

## CONCLUSIONS

This study examined the role of emotion dysregulation in ED outcome, and additionally, the contribution of self‐image of this association in a naturalistic sample of patients receiving specialized ED TAU. Results suggested emotion dysregulation as a potential mechanism of change rather than predictor in relation to outcome, where change in emotion regulation was associated with outcome at 1‐year follow‐up, and with change in ED psychopathology over the year. However, this association was mainly mediated by change in self‐image; relatively decreased self‐attack, ‐blame and/or ‐neglect and relatively increased self‐love, ‐affirmation and/or ‐protection was a mediator between improved emotion regulation and improved ED psychopathology.

Higher initial emotion dysregulation was weakly associated with higher follow‐up ED psychopathology, but this association did not remain when adjusting for initial clinical severity. Initial emotion dysregulation was not associated with diagnostic remission. These findings largely corroborate previous findings in BN/BED (Accurso et al., 2016; Anderson et al., 2020; MacDonald et al., 2017); initial emotion dysregulation may at best be an inconsistent, weak predictor of ED outcome. However, relative increase in emotion dysregulation, was strongly associated with both higher follow‐up ED psychopathology and increased risk of still having an ED, even when initial severity was adjusted for. As such, emotion dysregulation as a potential mechanism of change influencing ED outcome seems consistent regardless of diagnostic subgroup, treatment modality and treatment length (e.g. Hazzard et al., 2020; Peterson et al., 2017; Rowsell et al., 2016). About half of participants were in remission at 1‐year follow‐up with some diagnostic variation. Participants generally reduced their ED psychopathology over the year and improved in self‐image and emotion regulation. However, some participants reported higher levels of emotion dysregulation at follow‐up. Although most previous work suggest improvement in emotion dysregulation through treatment, there are reports of invariance, likely also including worsening (see review by Barney et al., 2019). This may reflect actual worsening during treatment, but other explanations may also be plausible. As suggested by Bruch (1978, 1982), emotional signalling in EDs may be misinterpreted or confused with other interoceptive sensations (e.g. hunger, satiety and tiredness), likely making emotionality, including emotion dysregulation ratings, particularly challenging in acute EDs. In contrast, ED symptoms and overall self‐blame, self‐attack and self‐neglect might be more clearly experienced and easier to verbalize, and thus easier to rate. Alternatively, due to the emotion regulating function of behavioural ED symptoms, some individuals may not experience their emotion regulation ability as deficient when presenting for treatment. However, with behavioural symptom abstinence, difficult emotions might resurface, leaving some feeling more emotionally helpless at follow‐up.

Although change in emotion dysregulation was strongly associated with ED outcome, results suggested that emotion regulation improvement was primarily associated with ED improvement *if* an improvement in the self‐image also occurred. Self‐image improvement on the other hand was strongly associated with ED improvement regardless of emotion dysregulation change. As such, these findings are consistent with previous findings highlighting the central role of self‐image in EDs (Forsén Mantilla & Birgegård, 2015); self‐directed evaluations and behaviours seem intertwined with the ED, potentially regardless of levels of other traits (i.e. such as emotion dysregulation) thought to influence symptoms. This, and prior cross‐sectional mediation results (Monell et al., 2015, 2020), may indicate emotion dysregulation as a more basic vulnerability factor in EDs. The results may also indicate that themes related to emotionality are experienced as distal and vague in relation to the more pressing self‐image and ED pathology. Self‐image may for instance be subjectively experienced as the constant internal ‘self‐talk’ regulating behaviour (‘I have to keep running’) and interpreting future, present and past events (‘of course they stared at me, I am so fat and ugly’). The more negative self‐image, the more negative and harsh tone of such thoughts. The mediation results may therefore describe how cognition (in the form of regulatory self‐reflection) represents emotion, whether elicited by internal or external stimuli. Put differently, the nature of one's self‐image influences on how one understands, tolerates, differentiates and represents emotions, and especially poorly integrated, confusing and frightening emotions associated with self‐destructive behaviours. When affected by an ED, these behaviours often include increased efforts to control dietary intake and one's body (e.g. Espeset et al., 2012). Self‐directed hate and blame may also translate into body‐directed hate and blame (I hate myself; therefore, I hate my body), enabling mistreatment, pushing and punishing of the body, but also providing some hope for change (‘if I exercise enough, I may be worthy of food, and eventually, of care and affection’).

Clinically, although increasing the ability to understand, tolerate, differentiate and manage emotions seems to increase the chance of ED remission, our results particularly highlight the importance of self‐image in this process. Working to improve a patient's self‐image may allow a person to develop a more adaptive inner working model that allows for the differentiation and integration of a wider range emotional experiences, thereby providing patients with necessary tools for reducing ED symptoms. This is not to say that standard ED interventions (i.e. restoring/stabilizing normal eating patterns) should be reduced, but our results indicate that emotional and self‐related processes may influence the success of symptom‐focused interventions and that helping patients to increasingly attend to themselves with acceptance rather than blame seems vital. An increased focus on psychological processes is also often requested by patients (Bezance & Holliday, 2013). Emotional and self‐related themes are specifically targeted within some ED treatments (e.g. dialectical behaviour therapy and compassion focused therapy for EDs, Enhanced CBT; Integrative Cognitive‐Affective Therapy; Berg & Wonderlich, 2013; Gale et al., 2014), but a therapeutic stance promoting better self‐image and thereby also addressing emotion regulation can be incorporated in most treatment modalities. EDs often entail interpersonal difficulties (e.g. conflicts, power struggles and social withdrawal), which may both aggravate and be aggravated by less beneficial aspects of self‐image. Creating a therapeutic climate where such themes can be openly discussed is therefore important, as is therapists being mindful of their actions towards the patient. In the case of a primarily negative self‐image, interpersonal interactions may tend to be interpreted as having hostile undercurrents (Benjamin, 2018). For instance, therapist friendly listening might be experienced as passive neglect, increasing the risk of patient walling off, potentially also reinforcing self‐neglect (‘no one cares about me, so why should I?’). Similarly, therapist positive protection (e.g. through renutrition interventions) may be experienced as intrusive negative control defensively reacted upon, and as such, patient and therapist may get stuck in negative control; others may experience interventions as critique, further reinforcing patient self‐blame (‘I’m never good enough, I let everybody down including my therapist’). Once self‐image has been better understood and begins to change, connections between difficult emotions and self‐directed behaviours might be easier to perceive. If there is more longstanding emotional vulnerability, being increasingly able to tolerate and accept emotional arousal, without going to harsh self‐blame and punishment, will likely have a beneficial effect on ED pathology. In a wider sense, being able to accept potential emotional (and other) vulnerabilities and taking better care of those traits by responding more compassionately and non‐judgementally to oneself, will likely entail less severe consequences of such vulnerabilities. Therefore, treatment that gradually increases the ability to treat oneself and one's emotions with acceptance rather than blame may be particularly important for improving overall mental health, reducing long‐term psychiatric vulnerability, and reducing the risk of ED relapse, although this remains to be examined.

### Strengths and limitations

Study strengths include the naturalistic sample, ensuring good ecological validity. Different diagnoses were represented, and patients received a variety of interventions, well reflecting the context for patients in specialized ED TAU. The study also investigated two relevant concepts and their interaction in relation to ED outcome, increasing specificity of proposed mechanisms.

However, several limitations need consideration. Considerable attrition at several stages potentially threatened sample representativeness. Although attrition analyses indicated overall patient‐related representativeness of the final sample (i.e. when compared to patients lacking ratings of emotion dysregulation and/or follow‐ups), unknown factors may still bias the sample. Also, among those with follow‐up assessments, those with DERS more often received psychotherapy, and among those, more often CBT compared to patients without initial DERS. This likely reflects therapist‐ rather than patient‐related factors, with psychotherapists (particularly CBT‐oriented) potentially being more interested in assessing additional traits. However, this likely had little impact on results, as there were no substantive differences between those groups. For analyses including change scores, patients with AN‐R initially rated lower levels of some emotion‐related scales. However, as this group only consisted 14% of the change analysis sample, this likely did not meaningfully affect overall results (if anything, this may have a mitigating influence due to floor effects). Our small sample size reduced power for associations with specific DERS subscales, and prevented analyses also considering pathology type/diagnosis, remission status, treatment modality and/or treatment length. Lastly, as we only had two time‐points, causality is still unclear why the temporal association chain of direct and indirect effects remains speculative. Although totally reversed causality is less likely, there may be feedback loops (e.g. more stable eating habits positively affecting emotion regulation and self‐image) not accounted for. However, this may primarily impact state—rather than trait effects; as emotion regulation and self‐image are processes that developed well ahead of EDs (Calkins et al., 2019), the hypothesized direction seems more plausible. Even so, future mediation analyses using additional time‐points could clarify temporal precedence, preferably including longer follow‐up times to examine potential associations with relapse. Further, more fine‐grained analyses including multiple mediators, patients with different clinical profiles and comorbid psychiatric symptoms, potentially also taking different treatment modalities and treatment length into account, would improve specificity and better reflect the clinical presentation of EDs.

### Final conclusions

Our results suggest that it is necessary to work with self‐image to improve emotion regulation and reduce ED symptoms. Initial emotion dysregulation seemed a poor predictor of outcome whereas improvement in emotion regulation was associated with better ED outcome at 1‐year follow‐up, and with ED psychopathology improvement over the year, suggesting that emotion dysregulation may be a potential mechanism of change influencing outcome. This association, in turn, was mainly mediated by improvement in self‐image, indicating that clinically, in order to improve emotion regulation as a means to reduce ED psychopathology, improving self‐image is essential.

## AUTHOR CONTRIBUTION

**Elin Monell:** Conceptualization; Data curation; Formal analysis; Methodology; Validation; Visualization; Writing—original draft; Writing—review & editing. **David Clinton:** Conceptualization; Supervision; Writing—original draft; Writing—review & editing. **Andreas Birgegård:** Conceptualization; Data curation; Methodology; Project administration; Resources; Supervision; Writing—original draft; Writing—review & editing.

## Data Availability

Data are not available for sharing due to ethical reasons; they also belong to the Stepwise registry.

## Footnotes

^1^
Excluding those who had not passed the follow‐up window.
