# Supplementary material for: Emotion dysregulation and eating disorder outcome: Prediction, change and contribution of self‐image
Source: Psychol Psychother. 2022 Mar 25;95(3):639–55. doi: 10.1111/papt.12391 (PMC9543735; doi:10.1111/papt.12391)
Supplement: Supplementary file 1 [file PAPT-95-639-s001.docx]

**SUPPLEMENTARY MATERIALS**

Emotion dysregulation and eating disorder outcome: prediction, change,

and contribution of self-image

Monell, E., Clinton, D., Birgegård, A. (2021)

| **Table S1.** Treatment and intervention information. *N*=302; due to unknown reasons, 5 patients with AN-R had completely missing data. | | | | | | | | | | | | | |
| --- | --- | --- | --- | --- | --- | --- | --- | --- | --- | --- | --- | --- | --- |
|  | | All | | AN-R | | AN-BP | | BN | | BED | | OSFED | |
|  | | *N*=302 | | *n*=45 | | *n*=15 | | *n*=104 | | *n*=19 | | *n*=119 | |
|  | | *N* (%) | | *n* (%) | | *n* (%) | | *n* (%) | | *n* (%) | | *n* (%) | |
| Treatment status | |  | |  | |  | |  | |  | |  | |
| Treatment terminated: | | 132 (44%) | | 13 (29%) | | 5 (33%) | | 62 (60%) | | 13 (68%) | | 39 (33%) | |
|  | *Completely terminated* |  | *108 (36%)* |  | *10 (22%)* |  | *5 (33%)* |  | *53 (51%)* |  | *10 (53%)* |  | *30 (25%)* |
|  | *Remaining supportive/low-frequency contact* |  | *24 (8%)* |  | *3 (7%)* |  | *0 (0%)* |  | *9 (9%)* |  | *3 (16%)* |  | *9 (8%)* |
| Treatment ongoing | | 170 (56%) | | 32 (71%) | | 10 (67%) | | 42 (40%) | | 6 (32%) | | 80 (67%) | |
| Additional treatment intensity | |  | |  | |  | |  | |  | |  | |
| Day-patient care | | 66 (22%) | | 11 (24%) | | 7 (47%) | | 18 (17%) | | 3 (16%) | | 28 (24%) | |
| Inpatient care | | 20 (7%) | | 9 (20%) | | 3 (20%) | | 2 (2%) | | 0 (0%) | | 6 (5%) | |
| Main treatment type(-s) | |  | |  | |  | |  | |  | |  | |
| Psychotherapy: | | 175 (58%) | | 20 (44%) | | 7 (47%) | | 66 (63%) | | 10 (53%) | | 72 (61%) | |
|  | *+ other interventions^*^ (incl. supportive therapy^***^)* |  | *121 (40%)* |  | *15 (33%)* |  | *5 (33%)* |  | *42 (40%)* |  | *5 (26%)* |  | *54 (45%)* |
|  | *Psychotherapy only* |  | *54 (18%)* |  | *5 (11%)* |  | *2 (13%)* |  | *24 (23%)* |  | *5 (26%)* |  | *18 (15%)* |
| Supportive therapy: | | 110 (36%) | | 21 (47%) | | 7 (47%) | | 31 (30%) | | 8 (42%) | | 43 (36%) | |
|  | *+ other interventions (excl. psychotherapy)* |  | *80 (26%)* |  | *14 (31%)* |  | *5 (33%)* |  | *25 (24%)* |  | *6 (33%)* |  | *30 (25%)* |
|  | *Supportive therapy only* |  | *30 (10%)* |  | *7 (16%)* |  | *2 (13%)* |  | *6 (6%)* |  | *2 (11%)* |  | *13 (11%)* |
| Other interventions only | | 17 (6%) | | 4 (9%) | | 1 (7%) | | 7 (7%) | | 1 (5%) | | 4 (3%) | |
| Type of psychotherapy | |  | |  | |  | |  | |  | |  | |
| CBT: | | 131 (75%) | | 13 (65%) | | 4 (57%) | | 53 (80%) | | 10 (100%) | | 51 (71%) | |
|  | *CBT only* |  | *114 (65%)* |  | *9 (45%)* |  | *4 (57%)* |  | *45 (68%)* |  | *9 (90%)* |  | *47 (65%)* |
|  | *CBT in combination with other psychotherapies^**^* |  | *17 (10%)* |  | *4 (20%)* |  | *0 (0%)* |  | *8 (12%)* |  | *1 (10%)* |  | *4 (6%)* |
| Other therapies^**^ (no CBT) | | 42 (24%) | | 6 (30%) | | 3 (43%) | | 12 (18%) | | 0 (0%) | | 21 (29%) | |
| Missing information | | 2 (1%) | | 1 (5%) | | 0 (0%) | | 1 (2%) | | 0 (0%) | | 0 (0%) | |
| ^*^Including psychoeducation, physical/dietary counselling, physiotherapeutic treatment, assisted eating, and/or other interventions. | | | | | | | | | | | | | |
| ^**^Including psychodynamic, systemic, interpersonal, dialectical behavior, and/or other psychotherapies. | | | | | | | | | | | | | |
| ^***^9 patients only received psychotherapy and supportive talks (i.e., no additional interventions). | | | | | | | | | | | | | |
| *Note:* AN-BP = anorexia nervosa binge/purge subtype; AN-R = anorexia nervosa restrictive subtype; BED = binge eating disorder; BN = bulimia nervosa; CBT = cognitive behavioral therapy; OSFED = other feeding and eating disorders. | | | | | | | | | | | | | |
